# Multimodal Therapy of Upper Gastrointestinal Malignancies

**DOI:** 10.3390/cancers13040793

**Published:** 2021-02-14

**Authors:** Ulrich Ronellenfitsch, Johannes Klose, Jörg Kleeff

**Affiliations:** Department of Visceral, Vascular and Endocrine Surgery, Martin-Luther-University Halle-Wittenberg, University Medical Center Halle (Saale), Ernst-Grube-Str. 40, 06120 Halle (Saale), Germany; johannes.klose@uk-halle.de (J.K.); joerg.kleeff@uk-halle.de (J.K.)

Upper gastrointestinal carcinomas comprise squamous cell carcinoma (SCC) and adenocarcinoma (AC) of the esophagus, as well as gastric AC. There are regionally different epidemiological trends for the diseases. While in many Asian countries, esophageal SCC and distal gastric AC are still the most common upper gastrointestinal carcinomas, their incidence has declined and the incidence of esophageal AC and proximal gastric AC has risen in many Western countries [1,2]. All of these tumor entities have in common that only multimodal treatment, i.e., a combination of chemotherapy, radiotherapy, and surgery tailored to the individual characteristics of the patient and the tumor, can achieve optimal outcomes and improve the hitherto often poor prognosis. The original research and review articles in this Special Issue of *Cancers* provide a comprehensive overview of the diagnosis—preoperative, surgical, postoperative—as well as systemic treatment for upper gastrointestinal carcinoma.

Correct and timely diagnosis is the foundation for any therapy. This holds particularly true for an oncological disease in which the exact tumor stage and histopathological tumor characteristics determine the appropriate treatment. As described in the reviews by Dumoulin et al. and Cummings et al. [3,4], high definition and virtual or dye chromoendoscopy can greatly enhance the diagnostic yield for early tumors. This holds particularly true in repeat endoscopy for Barrett’s esophagus and in screening for SCC, which is recommended in certain high-risk populations. Tumor stage should be ascertained by a combination of computed tomography (CT), endosonography, and, in selected patients, positron emission tomography-computed tomography (PET-CT).

While early esophageal and gastric cancers can be treated by advanced endoscopic techniques such as endoscopic submucosal dissection, there is now consistent evidence that for locally advanced cancers, neoadjuvant therapy is associated with survival benefits compared to surgery alone. For gastroesophageal AC, both neoadjuvant chemotherapy and chemoradiotherapy are viable options [5], with none of the two modalities having shown superiority over the other and randomized head-to-head comparisons still ongoing [6,7]. For esophageal SCC, neoadjuvant chemotherapy without radiotherapy has no role any longer, given the substantial survival benefit attained by neoadjuvant chemoradiotherapy [8]. The survival advantage could possibly be further augmented by administering intensified induction chemotherapy prior to chemoradiotherapy. This approach is used in ongoing trials comparing neoadjuvant chemoradiotherapy to chemotherapy for esophageal AC [6,7], but has also proven feasible and effective outside a population of selected trial participants, as shown in the retrospective study by Simoni et al. [9]. To achieve the best oncological outcomes, it is crucial to distinguish beforehand patients who will likely benefit from neoadjuvant therapy from those who will not. The histological subtype according to the Laurén classification might play an important role in this association. Schirren et al. showed that in patients with a diffuse type tumor, neoadjuvant chemotherapy is not associated with longer survival compared to surgery alone, as opposed to patients with intestinal- and mixed-type tumors, in whom neoadjuvant chemotherapy leads to a relevant survival benefit [10]. Patients might also be stratified according to HER2neu expression. A phase II/III trial comparing dual HER2neu blockade in combination with docetaxel, oxaliplatin, and fluorouracil/leucovorin (FLOT) versus FLOT alone for HER2-positive tumors showed promising results regarding histopathological response, which will now need to be corroborated regarding survival outcomes [11]. For esophageal SCC, following promising results and drug registration for unresectable tumors, neoadjuvant immune checkpoint inhibitor therapy is currently being tested in a phase III trial by the Japanese Esophageal Oncology Group, as described in the review article by Koyanagi et al. [12].

Schirren et al. demonstrated for gastroesophageal AC that histopathological response of the primary tumor, expressed in the proportion of remaining viable tumor cells, is an independent predictor of survival [13]. Besides, the histopathological TNM stage upon resection following neoadjuvant therapy has been shown to be predictive for survival [14,15]. A specific predictor could be the lymph node ratio, i.e., the number of metastatic lymph nodes divided by the number of lymph nodes harvested in total. Rawicz-Pruszyński et al. showed in their analysis that the ratio was inversely associated with survival in patients who had undergone neoadjuvant chemotherapy, with statistical significance being reached in the subgroups of patients with intestinal type tumors and those with no response of the primary tumor to neoadjuvant therapy [16].

Although postoperative mortality has substantially decreased in recent decades, as shown in a study by Galata et al. comprising gastrectomies conducted over a 40-year period, upper gastrointestinal cancer surgery still carries a relevant morbidity risk [17]. Preoperative risk stratification and preparation of patients is therefore important. Interestingly, pretherapeutic and preoperative sarcopenia measured by CT as one measure of nutritional status was not associated with postoperative complications in patients undergoing thoracoabdominal esophagectomy in a relatively small study by Grün et al. [18]. Yet, a potential effect of poor nutritional status in the cohort might have been offset by nutritional assessment and therapy, which was offered to all patients. This underlines the importance of appropriate prehabilitation prior to surgery for upper gastrointestinal cancers [19]. To achieve the best possible postoperative outcomes, both intra- and perioperative treatment are important. Technical details such as anastomotic techniques supposedly play a role. A retrospective study by Müller et al. found no association between the diameter of the stapler used for creating the anastomosis and the incidence of anastomotic leak and other complications [20]. Of note, in the study population, stapler size was chosen individually according to the size of the esophageal remnant. Therefore, the result is most likely rather an expression of the need for individually tailored surgical techniques than of the fact that technical details are of lower importance. Teoule et al. showed in their study that using a dedicated clinical pathway for patients undergoing oncological gastrectomy improved process quality outcomes, such as nutritional management and spirometer therapy, which are both known to be important contributors to postoperative recovery [21]. However, probably due to the relatively small study population, no significant effects on postoperative morbidity and mortality could be observed. An important factor contributing to fatal postoperative outcomes is the so-called failure to rescue, i.e., the inability to avert death in a patient suffering a postoperative complication. While the incidence of complications has remained unchanged, such failure has become less frequent in recent decades, probably due to advances in emergency perioperative care. This explains the decline in postoperative mortality shown by Galata et al. [17]. Averting postoperative mortality does not only have short-term effects. In the same cohort, patients who had survived a complication did not have different overall survival from those who did not suffer complications [22].

Although it has proven beneficial regarding long-term outcomes compared to surgery alone [23,24], postoperative chemotherapy or chemoradiotherapy has by now lost importance. It is generally aimed to administer these treatments preoperatively, especially if chemoradiotherapy is applied [8]. The standard chemotherapy schemes for gastroesophageal AC stipulate postoperative continuation of chemotherapy [25], but this requires a good and timely recovery from surgery. Given that this is not always feasible, usually only slightly more than half of patients proceed to the adjuvant part of chemotherapy. Adding postoperative radiotherapy to perioperative chemotherapy for gastric cancer has not been shown to increase survival and is thus not generally recommended [26,27]. Certain subgroups might still benefit from this approach. Yu et al. however failed to show in the population of the randomized ARTIST trial that a mesenchymal subtype (microsatellite-stable with epithelial-to-mesenchymal transition phenotype) was predisposed to higher susceptibility to postoperative chemoradiotherapy [28]. Adjuvant therapy does however have a stand in patients with an advanced histopathological tumor stage who were initially understaged and thus erroneously proceeded to upfront surgery. This once again emphasizes the importance of accurate pretherapeutic staging, as described above.

Treatment for metastatic or non-resectable upper gastrointestinal cancers poses a challenge, as described in the review by Accordino et al. [29]. First-line chemotherapy is usually platinum-based and consists of a doublet or triplet combination depending on the physical status of the patient. Some targeted therapies such as trastuzumab for HER2neu-overexpressing AC or ramucirumab for AC in second-line treatment are available. The immune checkpoint inhibitor nivolumab has recently been approved for second-line treatment of PD-L1 positive esophageal SCC, based on the results of a multi-national trial. For gastroesophageal AC, trials with immune checkpoint inhibitors have yielded heterogeneous results and no approval of a corresponding drug has been made so far. Other therapeutic approaches such as cancer vaccines or CAR-T cell therapy are still in early clinical testing. Surgery in metastatic disease might have a circumscribed role in gastroesophageal AC. A German phase II trial showed that oligometastatic patients, who proceeded to tumor resection after intensive chemotherapy, showed a favorable overall survival, almost reaching that of a non-metastatic control group [25]. The retrospective study by Choe et al. compared patients with oligometastatic disease undergoing resection of the primary and metastases to similar patients who did not proceed to surgery and found a survival benefit for the former [30]. Results from both studies are promising, but cannot be generalized because their non-randomized study designs were predisposed to selection bias. Therefore, the concept of surgical clearance of oligometastatic disease is currently being further tested and compared to non-surgical treatment in a randomized trial [31].

In summary, the articles in this Special Issue give proof of the many facets and challenges associated with upper gastrointestinal cancers. Both the patient and the tumor need to be assessed and treated individually and with a meaningful combination of the best available methods. Only by doing so, the prognosis of patients with these diseases can further be improved.
